# High-energy and low-cost membrane-free chlorine flow battery

**DOI:** 10.1038/s41467-022-28880-x

**Published:** 2022-03-11

**Authors:** Singyuk Hou, Long Chen, Xiulin Fan, Xiaotong Fan, Xiao Ji, Boyu Wang, Chunyu Cui, Ji Chen, Chongyin Yang, Wei Wang, Chunzhong Li, Chunsheng Wang

**Affiliations:** 1 Department of Chemical and Biomolecular Engineering, University of Maryland, College Park, MD USA; 2 Department of Chemical Engineering, East China University of Science and Technology, Shanghai, China; 3 School of Materials Science and Engineering, East China University of Science and Technology, Shanghai, China; 4 Energy & Environment Directorate, Pacific Northwest National Laboratory, Richland, WA USA

**Keywords:** Batteries, Energy grids and networks

## Abstract

Grid-scale energy storage is essential for reliable electricity transmission and renewable energy integration. Redox flow batteries (RFB) provide affordable and scalable solutions for stationary energy storage. However, most of the current RFB chemistries are based on expensive transition metal ions or synthetic organics. Here, we report a reversible chlorine redox flow battery starting from the electrolysis of aqueous NaCl electrolyte and the as-produced Cl_2_ is extracted and stored in the carbon tetrachloride (CCl_4_) or mineral spirit flow. The immiscibility between the CCl_4_ or mineral spirit and NaCl electrolyte enables a membrane-free design with an energy efficiency of >91% at 10 mA/cm^2^ and an energy density of 125.7 Wh/L. The chlorine flow battery can meet the stringent price and reliability target for stationary energy storage with the inherently low-cost active materials (~$5/kWh) and the highly reversible Cl_2_/Cl^−^ redox reaction.

## Abstract (web summary)

Flow batteries provide promising solutions for stationary energy storage but most of the systems are based on expensive metal ions or synthetic organics. Here, the authors show a chlorine flow battery capitalizing the electrolysis of saltwater where the redox reaction is stabilized by the saltwater-immiscible organic flow.

## Introduction

Integrating renewable energy, such as solar and wind power, is essential to reducing carbon emissions for sustainable development. However, large-scale utilization is hindered by the intermittence and uneven distribution of these power sources^1–3^. Implementation of grid-scale energy storage is essential to mitigate the mismatch between electricity production and consumption^4^. Different technologies are developed for this purpose, including supercapacitors, sodium–sulfur batteries, pump hydro, flywheels, and superconducting magnetic energy^5^. Redox flow battery (RFB) is considered one of the most attractive energy storage systems for large-scale applications due to the lower capital cost, higher energy conversion efficiency, and facile modularity^6,7^. The cores of flow cells are the circulating electrolytes that carry the redox-active materials for energy storage and release.

Currently, the all-vanadium RFB is the most researched and developed RFB chemistry; however, the market adoption of this system has been hampered by high-cost chemicals (material cost close to 60% of the overall system cost^8^ and low energy density. Although aqueous soluble organic redox species offer a potential option for low-cost materials^9–15^, the synthetic processes required to customize the molecular structure for high solubility and optimal potential will again limit the material cost and availability^6,16–18^. Also, they rely on the costly ion-permeable membranes to reduce cross-over, further increasing capital and maintenance costs^19^.

Recently, polymer redox couples were developed to circumvent ion-permeable membranes^20^, and the semi-solid Li-ion (suspensions of Li-ion battery active materials in nonaqueous electrolytes) systems have been explored for higher energy density and efficiency. However, high viscosity, lower peak power operation time, and high material cost emerged with these systems^4,21,22^.

To meet the needs of RFB chemistries with the naturally abundant and low-cost redox-active materials, we report a new RFB system that capitalizes the electrolysis of saltwater or aqueous NaCl electrolyte using the Cl_2_/Cl^−^ redox couple as the active material for the positive electrode. The Cl_2_/Cl^−^ has a theoretical capacity of 755 mAh/g, more than two times that of vanadium oxides (VO_2_^+^/VO^2+^, 226 mAh/g) used in current RFBs. Cl_2_/Cl^−^ redox chemistry is a fast single-electron transferred reaction with an activation energy of 35.5 kJ/mol^23,24^, which is comparable to or even smaller than that of VO_2_^+^/VO^2+^^25^, thus is suitable for high power applications. In addition, sodium chloride is one of the cheapest commodities available due to the abundant source in seawater and large-scale production (~$40 per metric ton)^26,27^. These features enable Cl_2_/Cl^−^ redox reaction to be a promising candidate for RFB.

Rarely heard in the battery history is that Cl_2_/Cl^−^ redox couple was used in the RFB to power the first fully controlled airship La France in 1884^28^. The Cl_2_/Cl^−^ based batteries are often typified by low Coulombic efficiency (CE) of 40–70%^29–33^ due to Cl_2_ dissolution in the electrolytes and large voltage hysteresis (0.7 V at 32 mA/cm^2^) due to non-wettability between electrolytes and electrodes^34,35^, which limits the energy efficiency to around 60%. Graphite was reported as chlorine storage host via intercalation^36^. However, the instability of Cl_2_ intercalated graphite at room temperature results in low storage capacity (35–40 mAh/g) and limited cycle life. After that, no other materials with appropriate stability, storage capacity, and reaction kinetics have been reported to enable reversible Cl_2_ electrochemical reaction.

Our objective is to develop a new RFB with the highly reversible Cl_2_/Cl^−^ redox species through electrolyzing the saturated NaCl aqueous electrolyte (NaCl/H_2_O) and storing the as-produced Cl_2_ in water-immiscible organic phases such as carbon tetrachloride (CCl_4_) or mineral spirits. These organic phases provide several desirable properties: (1) Cl_2_ in CCl_4_ (Cl_2_-CCl_4_) delivers a volumetric capacity of 97 Ah/L due to high solubility of Cl_2_ in CCl_4_ (0.184 mole/mole CCl_4_^37^, which is a 2 to 4 times improvement over the current vanadium-based catholyte (22.6–43.1 Ah/L^38^; (2) The Cl_2_-CCl_4_ is immiscible to NaCl/H_2_O, thus requires no membrane to prevent cross-over, further reducing costs; (3) The Cl_2_-CCl_4_ has low and constant viscosity of 0.819 mPa.s, in contrast to high and varying viscosity of aqueous vanadium-based catholyte (1.4–3.2 mPa.s^39^, thus is easy to flow; (4) Cl_2_-CCl_4_ can wet carbon porous electrodes easily, which significantly enhances the surface area for Cl_2_ storage and reaction; (5) Cl_2_ has high diffusivity in CCl_4_, minimizing energy dissipation for mass transport.

## Results

### Storage and electrochemical performance of Cl_2_-CCl_4_

**Fig. 1:**
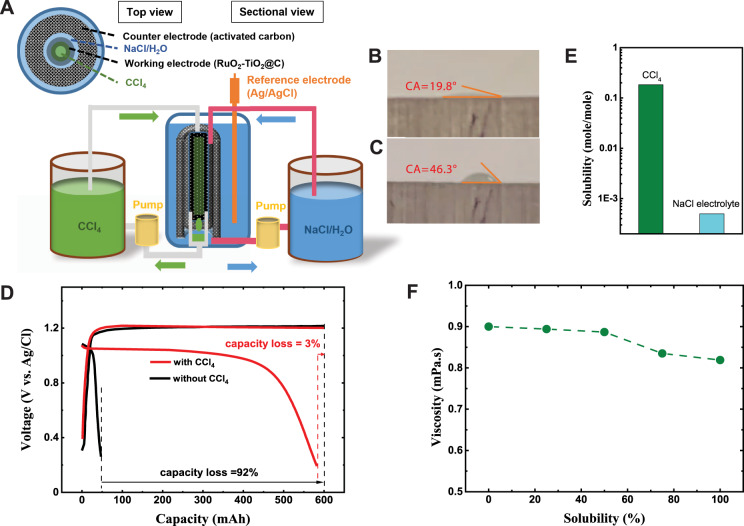
Electrochemical performance and physical properties of Cl_2_-CCl_4_. **A** Schematic of the three-electrode cell. Inset shows the cylindrical structure of the cell from the top view, in which the inner diameter of the RuO_2_-TiO_2_@C working electrode is 2.0 mm, the thickness of the RuO_2_-TiO_2_@C electrode is 1.0 mm, the distance between the working and counter electrode is 3.0 mm and the thickness of the counter electrode is 3.0 mm. The height is 2.0 cm, and the volume capacity of the cell is around 2.0 mL. The total volumes of the CCl_4_ reservoir and the NaCl/H_2_O reservoir are 6.0 mL and 2.0 mL, respectively. **B** CA of CCl_4_ on graphite plate electrode. **C** CA of NaCl/H_2_O on graphite plate electrode. **D** Galvanostatic charge and discharge profiles of Cl_2_-CCl_4_ (red) and Cl_2_ without CCl_4_ (black) at the current density of 20 mA/cm^2^. Both cells ran with constant charge capacity of 600 mAh at Q_aq_ (flow rate of NaCl/H_2_O) = 0.02 mL/s and Q_org_ (flow rate of CCl_4_) = 0.002 mL/s. The differences between discharge and charge capacity are labeled as percentage capacity loss. **E** The solubility of Cl_2_ in CCl_4_ and NaCl/H_2_O. **F** The viscosities of Cl_2_-CCl_4_ with different concentrations of Cl_2_ (100% refers to saturation).

The Cl_2_/Cl^−^ redox reaction in NaCl/H_2_O was evaluated in a concentric cell with RuO_2_-TiO_2_ coated porous carbon (RuO_2_-TiO_2_@C) as a working electrode, activated carbon as a counter electrode (Fig. S1), and Ag/AgCl as the reference electrode (Fig. 1A). The RuO_2_-TiO_2_ catalysts on porous carbon (Figs. S2, S3) are used to promote the oxidation kinetics of chloride^40–43^ (Fig. S4). CCl_4_ was pumped through the working electrode, and the NaCl/H_2_O through the interstitial space between the working and counter electrodes to ensure adequate Cl^-^ supply. While CCl_4_ and NaCl/H_2_O entered the RuO_2_-TiO_2_@C electrode as separate flows, they both wet the carbon electrode, demonstrated by <90° contact angles (CAs) on a graphite plate electrode (Fig. 1B, C). And the two liquids take up 66.2% and 33.8% of the void volume in the RuO_2_-TiO_2_@C electrode, respectively (see the determination of percentage volume in Supplementary Note 1). The ion-permeable membrane used in traditional RFBs to prevent cross-contamination^15,16,44–46^ is not required here since the Cl_2_-CCl_4_ and NaCl/H_2_O are phase separated.

During charge, the Cl_2_ was generated from oxidizing the Cl^−^ in the RuO_2_-TiO_2_@C electrode. The reaction shows a constant potential at 1.2 V versus Ag/AgCl reference electrode [1.36 V versus normal hydrogen electrode (NHE)]. During discharge, the Cl_2_ in CCl_4_ was reduced to Cl^−^ in the working electrode and entered the NaCl/H_2_O (see the formulation for positive electrode reaction). The presence of CCl_4_ flow significantly enhances the coulombic efficiency (CE) from 8 to 97% (Fig. 1D). Because the solubility of Cl_2_ in CCl_4_ is three orders of magnitude higher than that in NaCl/H_2_O (0.184 mole/mole CCl_4_ versus 0.0005 mole/mole NaCl/H_2_O^38^) (Fig. 1E), the Cl_2_ generated during the charging process can be stored in CCl_4_, which prevents Cl_2_ diffusion into NaCl/H_2_O as supported by Raman spectroscopy (Fig. S5) and the positive Gibbs free energy to transfer Cl_2_ from CCl_4_ to NaCl/H_2_O (Fig. S6). When 6.0 mL CCl_4_ was used, a maximum reversible capacity for Cl_2_/Cl^−^ conversion is 600 mAh (Fig. S7), rendering the capacity of 97 Ah/L for the Cl_2_-CCl_4_.

#### Positive electrode reaction

$$\begin{array}{cc}2{{{{{{\rm{Cl}}}}}}}^{-}\,-\,2{{{{{{\rm{e}}}}}}}^{-}\leftrightarrow {{{{{{\rm{Cl}}}}}}}_{2} & \quad{E}^{0}\,=\,1.36\,{{{{{\rm{V}}}}}}({{{{{\rm{versus}}}}}}\,{{{{{\rm{NHE}}}}}})\end{array}$$

The Cl_2_-CCl_4_ positive electrode has a low and almost consistent viscosity. When the concentration of Cl_2_ increases from 0 to 0.184 mole/mole CCl_4_ (saturation), the viscosity even slightly decreases from 0.894 to 0.819 mPa.s (Fig. 1F) in accord to Eyring’s absolute reaction rate theory for gas–liquid mixtures^47,48^. On the other hand, the viscosity of common catholyte could increase by several or even dozen times as the concentration of solute increases^49^. The low viscosity of Cl_2_-CCl_4_ reduces the pumping loss^40^, and the steady viscosity minimizes the volumetric transfer between catholyte and anolyte at different SOCs^50,51^.

### Full chlorine flow battery (CFB)

**Fig. 2:**
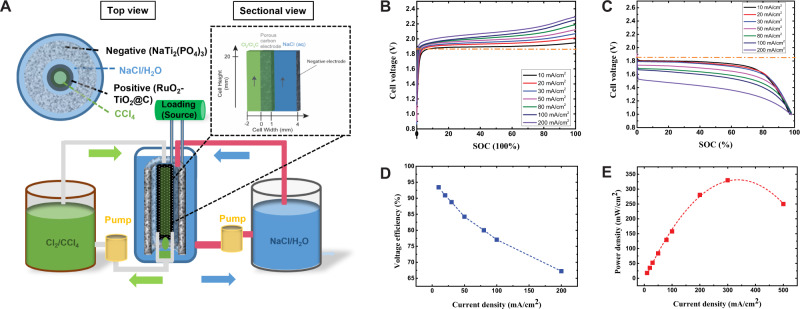
Schematic and electrochemical performance of chlorine flow battery (CFB). **A** Schematic of the CFB, the inner diameter of the tube containing CCl_4_ and RuO_2_-TiO_2_@C electrode is 2.0 mm, the thickness of the RuO_2_-TiO_2_@C electrode is 1.0 mm, the distance between the working and counter electrode is 3.0 mm. The thickness of the counter electrode is 3.0 mm. The height of the cell is 2.0 cm, and the volume capacity of the cell is around 2.0 mL. The total volumes of the CCl_4_ reservoir and the NaCl/H_2_O reservoir are 6.0 mL and 2.0 mL, respectively. Q_aq_ = 0.02 mL/s and Q_org_ = 0.002 mL/s. Galvanostatic charge **B** and discharge **C** profiles of the CFB at different current densities. The state of charge (SOC) of the battery is normalized to the maximum reversible capacity at 10 mA/cm^2^, in which 100% SOC represents charge to 600 mAh. **D** The voltage efficiencies of the CFB at different current densities. **E** The power densities of the CFB at different current densities.

To fabricate a full CFB, the activated carbon counter electrode was replaced by NaTi_2_(PO_4_)_3_ negative electrode (Fig. 2A). NaTi_2_(PO_4_)_3_ (Figs. S8, S9) was chosen as the negative electrode due to low potential (−0.5 V (versus NHE), rapid and reversible Na-ion insertion/extraction in NaCl/H_2_O demonstrated by the symmetric anodic and cathodic peaks with 60 mV separation in the cyclic voltammetry (negative electrode reaction and Fig. S10A)^52^. The NaTi_2_(PO_4_)_3_ shows a 65% capacity retention even at the C-rate of 315 C (1 C = fully discharge/charge within 1 hour, Fig. S10) and long cycle life of 1000 cycles (Fig. S11).

#### Negative electrode reaction

$$\begin{array}{cc}{{{{\rm{Na}}}}}_{3}{{{{\rm{Ti}}}}}_{2}{({{{{\rm{PO}}}}}_{4})}_{3}\,-\,2{{{{\rm{e}}}}}^{-}\,-\,2{{{{\rm{Na}}}}}^{+}\leftrightarrow{{{{\rm{NaTi}}}}}_{2}{({{{{\rm{PO}}}}}_{4})}_{3} & {{{{\rm{E}}}}}^{0}\,=\,-0.5\,{{{\rm{V}}}}({{{\rm{versus}}}}\,{{{\rm{NHE}}}})\end{array}$$

While the overpotentials enhanced (orange dash lines in Fig. 2B, C) as the current density increased, the discharge capacities did not vary (Fig. 2B, C), which could be attributed to the large reaction surface area endowed by the wetting between carbon electrode and Cl_2_-CCl_4_ (Fig. 1B, C). Fig. 2D demonstrates cell voltage efficiency (defined as the potential ratio of discharge to charge) of 93.6% at 10 mA/cm^2^ and ~77% at 100 mA/cm^2^. The multiplication of discharge capacity and voltage gives the cell power density that peaks at 325 mW/cm^2^ when operated at 350 mA/cm^2^ (Fig. 2E). It is worth noting that polarizations for discharge are more significant than those for discharge (Fig. 2B, C). In the CFB, overpotentials are caused by redox reactions and concentration gradient. Since the symmetric factors for Cl^−^/Cl_2_ redox reactions are equal^17,25^, the overpotentials needed to drive the reduction and oxidation reaction are the same, the different overpotentials for charge and discharge observed here could only be attributed to the concentration gradient.

**Fig. 3:**
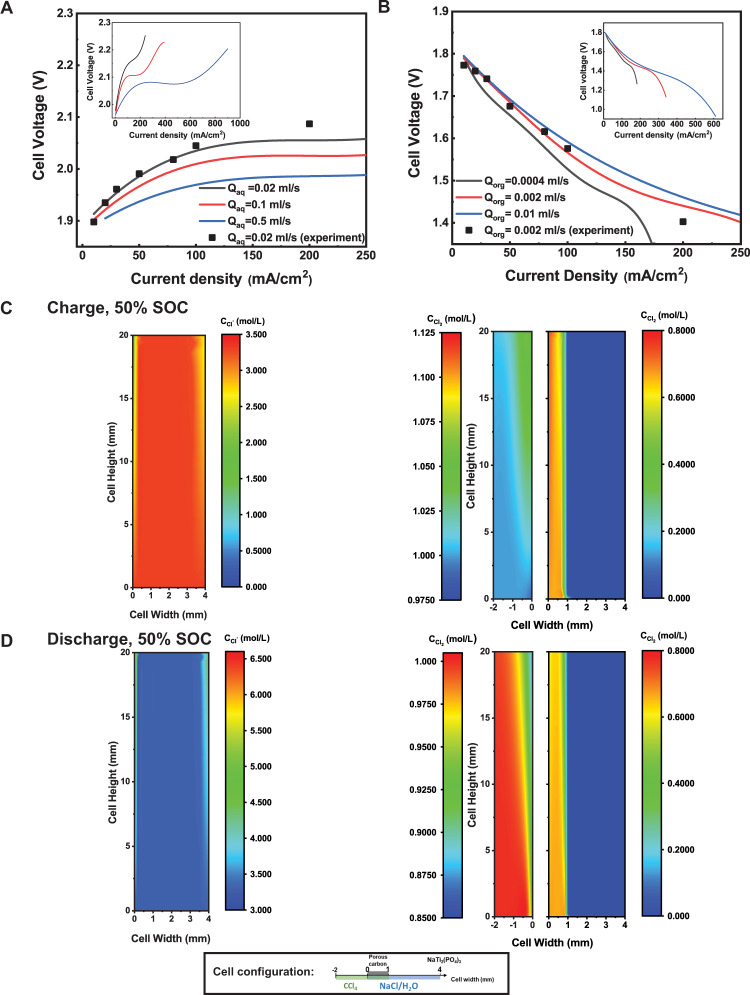
Simulation of the CFB. **A** Steady-state potentials of CFB charged at 50% SOC with different Q_aq_ and Q_org_ = 0.002 mL/s, inset shows the whole current density range demonstrating steady charge potential. **B** Steady-state potentials of CFB discharged at 50% SOC with different Q_org_ and Q_aq_ = 0.02 mL/s, inset shows the whole current density range demonstrating steady discharge potential. **C** Distribution of Cl^−^ and Cl_2_ in the CFB charged at 50% SOC and 50 mA/cm^2^ with Q_aq_ = 0.02 mL/s and Q_org_ = 0.002 mL/s. **D** Distribution of Cl^−^ and Cl_2_ in the CFB discharged at 50% SOC and 50 mA/cm^2^ with Q_aq_ = 0.02 mL/s and Q_org_ = 0.002 mL/s. The position of Cl_2_-CCl_4_, NaCl/H_2_O, porous RuO_2_-TiO_2_@C positive electrode and NaTi_2_(PO_4_)_3_ negative electrode are labeled in the legend.

A steady-state model was developed to understand the species distribution and controlling steps in the CFB. The Nernst-Plank equation was applied to the porous RuO_2_-TiO_2_@C electrode (cell width = 0–1.0 mm in Fig. 2A), and NaCl/H_2_O (cell width = 1.0–4.0 mm in Fig. 2A), Fick’s equation was applied to the Cl_2_-CCl_4_ phase (cell width = −2.0–0 mm in Fig. 2A). The negative electrode was involved implicitly at the boundary of the NaCl/H_2_O (cell width = 4.0 mm in Fig. 2A) (see model description and Tables S1–S4 in Supplementary Note 1). The model was validated by the agreement between the simulated and experimental cell voltages (black lines and dots in Fig. 3A, B, experimental potential retrieved from Fig. 2B, C) at the same flow rates and current densities.

The model was then used to visualize the species distribution in the NaCl/H_2_O and in Cl_2_-CCl_4_. During charge, the Cl^−^ in NaCl/H_2_O was consumed inside the porous carbon electrode (Fig. 3C) and limits the reaction kinetics; during discharge, Cl_2_ in CCl_4_ is consumed and limits the reaction kinetics. The Cl^−^ concentration gradients are more significant than the Cl_2_ concentration gradient in the porous electrode for both charge and discharge (Fig. 3C, D), which is the result of a smaller diffusivity of Cl^−^ (1.5 × 10^−5^ cm^2^/s for Cl^−^, 2.0 × 10^−5^ cm^2^/s for Cl_2_ in NaCl/H_2_O and 3 × 10^−5^ cm^2^/s for Cl_2_ in CCl_4_^53–55^ and lower volume percentage of NaCl/H_2_O than CCl_4_ in the porous carbon electrode. The distinct species that control charge and discharge kinetics thus generate the asymmetric charge and discharge overpotentials (Fig. 2B, C). Since Cl^−^ and Cl_2_ are in different phases, increasing the flow rate of NaCl/H_2_O during charge and that of the Cl_2_-CCl_4_ during discharge enhance the mass transport of the limiting species accordingly, in which not only the overpotentials reduce, but the current density range allowing steady cell voltage extends (inset of Fig. 3A, B). At the highest flow rate examined, the voltage efficiency could be postulated to >93% at 20 mA/cm^2^.

**Fig. 4:**
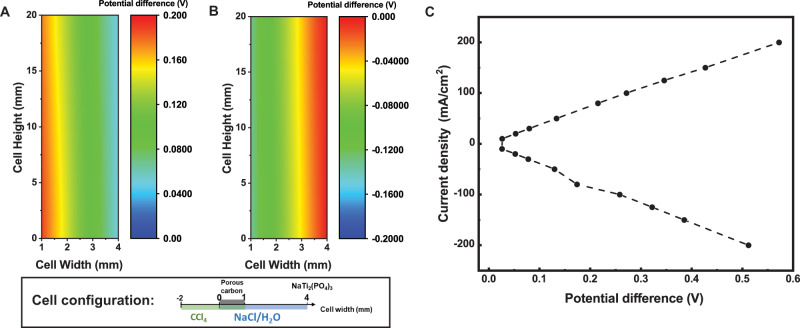
Potential gradient in the electrolyte of CFB. **A** Potential distribution during charge and **B** during discharge at 50% SOC and 50 mA/cm^2^. **C** The potential loss due to ion transport in the NaCl/H_2_O at different current densities. In all cases Q_aq_ = 0.02 mL/s and Q_org_ = 0.002 mL/s. The positions of Cl_2_-CCl_4_, NaCl/H_2_O, porous RuO_2_-TiO_2_@C positive electrode, and NaTi_2_(PO_4_)_3_ negative electrode are labeled in the legend.

The high voltage efficiency of the cell is attributed not only to the fast reaction kinetics but also the membrane-free configuration. The potential gradient in the NaCl/H_2_O was determined by the model (Fig. 4A, B), and the potential difference across the cell at half-cell height was plotted. The potential drop of ~20 mV at 10 mA/cm^2^ and ~250 mV at 100 mA/cm^2^ (Fig. 4C) are equivalent to proton transport but over 5 times smaller than Na^+^ and K^+^ transport in Nafion ion-permeable membranes in aqueous flow batteries with similar cell dimensions^56^. Thus, removing the ion-selective membrane opens a range of chemistries to be investigated, as the charge carriers can be chosen arbitrarily.

**Fig. 5:**
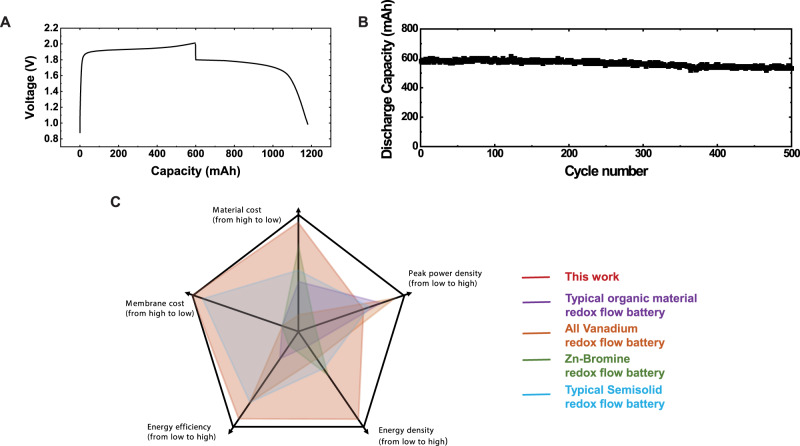
Charge and discharge behavior of CFB and comparisons of the performance matrices to redox flow batteries reported in the past 10 years. **A** Cell voltage profiles during constant-current cycling and **B** cycle performance of CFB at 20 mA/cm^2^, Q_aq_ = 0.02 mL/s and Q_org_ = 0.002 mL/s, and the charge capacity was set to be 600 mAh. The amount of CCl_4_ is 6.0 mL, the size of the porous RuO_2_-TiO_2_@C electrode is 1.0 mm-thick and 2.0 cm^2^ area. **C** The comparison of performance matrices among CFB, organic redox flow battery (anthraquinones as the anode material and ferricyanide as cathode material, ref. S24), all**-**vanadium redox flow battery (refs. S28, 29), Zn-Bromine redox flow battery (ref. S33), and semi**-**solid redox flow battery (Li as the anode and LiFePO_4_ as cathode material ref. S34) (see details in Table S5).

The CFB demonstrates the round-trip energy efficiency of 91% (calculated by voltage efficiency × Coulombic efficiency) at 10 mA/cm^2^ and provides an energy density of 125.7 Wh/L (see Methods), which is among the highest of the flow battery systems reported in past 10 years (Table S5). It is worth noting that the Cl_2_-CCl_4_ is different from bromine used in flow batteries that faces the serious self-discharge due to the diffusion of Br_2_ to the negative electrodes in the form of polybromide. When ion-permeable membranes were used to decrease Br_2_ cross-over, voltage efficiency was significantly limited by the transport of ions in the membrane, resulting in <80% energy efficiency in overall performance^57–59^. Figure 5A, B show the measured cell voltage profile and stable round-trip cycling for this battery at 20 mA/cm^2^ with a charge storage capacity of 600 mAh and the stable capacity retention for 500 cycles.

## Discussion

In this study, CCl_4_ was used as a proof of concept, it can be replaced by other liquids with high Cl_2_ solubility and are immiscible with NaCl/H_2_O. The candidates include heptane (chlorine solubility = 0.173 mole fraction at ambient temperature), octane (chlorine solubility = 0.168 mole fraction at ambient temperature), tetradecane (chlorine solubility = 0.254 mole fraction at ambient temperature)^29^ and mineral spirit. Mineral spirit demonstrates good wettability (CA = 9.1°) with carbon current collector (Fig. S12A), low viscosity (1.24 mPa.s), low toxicity, and is cheaper than CCl_4_^60^. When CCl_4_ was replaced by mineral spirit in the CFB, a volumetric capacity of 91.6 Ah/L was delivered at 20 °C (Fig. S12B).

The removal of the ion-permeable membrane also allows multivalent ions as charge carriers. When ZnCl_2_ is added to the electrolyte, NaTi_2_(PO_4_)_3_ can be replaced by zinc metal electrode, increasing the cell operating voltage to 1.9 V (Fig. S13).

Cost is one of the significant concerns to implementing flow batteries on a large scale for stationary energy storage. Considering that the ion-permeable membrane (mainly perfluorinated polymers) takes up more than 30% of the cost of flow batteries, significant cost reduction is expected with the membrane-free design^20^_._ The total material cost for energy storage with the proposed CFB is estimated to be ~$5/kWh, which is the cheapest among all the current flow battery systems (Fig. 5C and Table S5). In addition, the RuO_2_ catalyst for chlorine evolution reaction (CER) can also be replaced by tin, zinc, cobalt, and other cheap metal oxides partially^30^. Therefore, the proposed CFB design leaves significant space to meet the stringent target of ~$100/kWh for RFB applications^61^.

Cl_2_ is a reactive chemical commodity used in paper, plastic, dye, textile, medicine, antiseptics, insecticide, solvent, and paint industries. Administration and engineering controls for storage and transport are available to confine the incident rate to 0.019% of total chlorine shipments between 2007 and 2017^62^. The Occupational Safety and Health Administration of the United States has set a permissible exposure limit at a time-weighted average of 0.1 ppm (0.68 mg/m^3^) for bromine, 0.05 mg/m^3^ for vanadium pentoxide dust, 0.1 ppm (0.4 mg/m^3^) for quinone, and 1.0 ppm (3 mg/m^3^) for chlorine^63^. Thus, there is no apparent increase in chemical exposure risk when changing to chlorine redox reaction.

However, protections and cautions are still crucial. The CFB proposed here is a closed system in which the leakage of Cl_2_ gas is minimized by the fluoropolymer gasket (see Supplementary Note 2 for evaluation of chlorine permeation). Strategies from the chloro-alkali industry can be applied to reduce the risk of exposure upon scaling up, such as external seal pipe, shutoff system, neutralization reagents (scrubber)^64^, and sensing systems^65^.

In summary, the CFB proposed has demonstrated several unique advantages over current flow battery systems, including higher energy density, higher round-trip energy efficiency, and significantly lower prices. The membrane-free design enables both anionic and cationic charge carriers for a RFB, thus expanding the material and chemistry space of the redox flow technologies.

## Methods

### Material synthesis

The activated carbon with RuO_2_/TiO_2_ particles was prepared by dissolving 0.69 mmol RuCl_3_ and 1.622 mmol C_16_H_36_O_4_Ti in 100 mL isopropanol, then adding 2.0 g activated carbon into the solution. The mixture was stirred for 2 hours, and then the isopropanol was evaporated at 90 °C. Finally, the products were annealed at 500 °C for 1 hour under ambient conditions.

The carbon-coated NaTi_2_(PO_4_)_3_ was synthesized from 0.002475 mol Na_2_CO_3_, 0.01485 mol NH_4_H_2_PO_4_, and 0.0099 mol TiO_2_ in 100 mL of a 2.0 wt% poly-vinyl-alcohol (PVA) aqueous solution. The mixture was stirred at 80 °C until the water evaporated and white solids formed. The white solids were placed in a porcelain boat and heated at 900 °C for 10 hours with an increasing rate of 5 °C/min under an N_2_ flow in a tube furnace. To improve the cycling stability and electronic conductivity, thermal vapor deposition (TVD) was employed to prepare carbon-coated NaTi_2_(PO_4_)_3_ after calcination. The as-prepared powder was transferred into a reaction tube to make a fluid-bed layer for the reaction at 700 °C for 2 hours where a toluene vapor was carried by N_2_ through the reaction tube at a flow rate of 1 L/min, followed by heat-treatment at 900 °C for 2 hours without toluene carrying gas to increase its electronic conductivity. The temperature increasing rate is 5.0 °C/min.

### Electrode preparation and electrochemical measurements

The working electrode was fabricated by pressing a mixture of the active materials (porous carbon or carbon-coated NaTi_2_(PO_4_)_3_), carbon black, and PTFE (polytetrafluoroethylene) binder at the weight ratio of 7:2:1 onto a titanium grid with a pressure of 10 MPa. The cyclic voltammograms (CV) were obtained using a three-electrode cell with an active carbon counter electrode and Ag/AgCl reference electrode (0.197 V versus NHE). In the concentric cell, the inner diameter of the tube containing CCl_4_ and the RuO_2_-TiO_2_@C electrode is 2.0 mm, the thickness of the porous carbon electrode is 1.0 mm, the distance between the counter and working electrodes is 3.0 mm, and the thickness of the counter electrode is 3.0 mm. The height of the cell is 2.0 cm, and the volume capacity of the cell is around 2.0 mL. The total volume of the CCl_4_ reservoir is 6.0 mL, and the total volume of the NaCl/H_2_O reservoir is 2.0 mL. The CV measurements were carried out on a CHI660B electrochemical workstation. The galvanostatic charge and discharge profiles were obtained with an Arbin battery test station.

### Material characterizations

Scanning electron microscopy (SEM) images were taken with Hitachi SU-70 analytical SEM (Japan). Powder X-ray diffraction (PXRD) data were collected on a Bruker D8 X-ray diffractometer using Cu Kα radiation (*λ* = 1.5418 Å). Raman spectroscopy was performed on a Horiba Jobin Yvon Labram Aramis using a 532 nm diode-pumped solid-state laser, attenuated to give ~900 μW power at the sample surface. Viscosity Measurements were carried out using a CANNON-FENSKE viscometer.

### Energy density calculations

The energy density of CFB was calculated based on the 600 mAh cell used in this study with Eq. (1). The average operating potential is 1.8 V at 10 mA/cm^2^, the volume of CCl_4_ is 6.0 mL, the volume of NaCl/H_2_O is 2.0 mL and the volume of Na(Ti_2_(PO_4_)_3_) is 0.592 mL (weight = 5.0 g, density = 2.96 g/mL, volume = 2.96 g/mL ÷ 5.0 g = 0.592 mL). The total volume of active materials is 8.592 mL. Based on these configurations, the cell-level energy density (based on active materials) is 125.7 Wh/L.

1 $${{{{{\rm{Energy}}}}}}\,{{{{{\rm{density}}}}}}\,=\,\frac {{{{{{\rm{Cell}}}}}}\,{{{{{\rm{ capacity}}}}}}\,\times\, {{{{{\rm{average}}}}}}\,{{{{{\rm{potential}}}}}}}{{{{{{\rm{Total}}}}}}\ {{{{{\rm{volume}}}}}}\,{{{{{\rm{of}}}}}}\,{{{{{\rm{active}}}}}}\,{{{{{\rm{materials}}}}}}}$$

## Supplementary information

Supplementary Information

Peer Review File

## Data Availability

The data that support the findings within this paper are available within the article and Supplementary Information. Additional data are available from the corresponding authors upon request. Source data are provided with this paper.

## Source data

Source Data
