# Supplementary material for: High-energy and low-cost membrane-free chlorine flow battery
Source: Nat Commun. 2022 Mar 11;13:1281. doi: 10.1038/s41467-022-28880-x (PMC8917161; doi:10.1038/s41467-022-28880-x)
Supplement: Supplementary file 1 — Supplementary Information [file 41467_2022_28880_MOESM1_ESM.pdf]

Supplementary Information for

High Energy and Low-Cost Membrane-free Chlorine flow Battery

Singyuk Hou[1†], Long Chen[1,2†*], Xiulin Fan[1†], Xiaotong Fan[3], Xiao Ji[1], Boyu Wang[1], Chunyu Cui[1], Ji Chen[1], Chongyin Yang[1], Wei Wang[4], Chunzhong Li[2], Chunsheng Wang[1*]

[1]Department of Chemical and Biomolecular Engineering, University of Maryland, College Park, MD 20742, USA.

[2] Department of Chemical Engineering, East China University of Science and Technology, Shanghai, China.

[3] School of Materials Science and Engineering, East China University of Science and Technology, Shanghai, China.

[4]Energy & Environment Directorate, Pacific Northwest National Laboratory, Richland, WA, USA

[†] These authors contributed equally: Singyuk Hou, Long Chen, and Xiulin Fan

*Email: longchen@ecust.edu.cn, cswang@umd.edu

Supplementary Notes 1

Model description

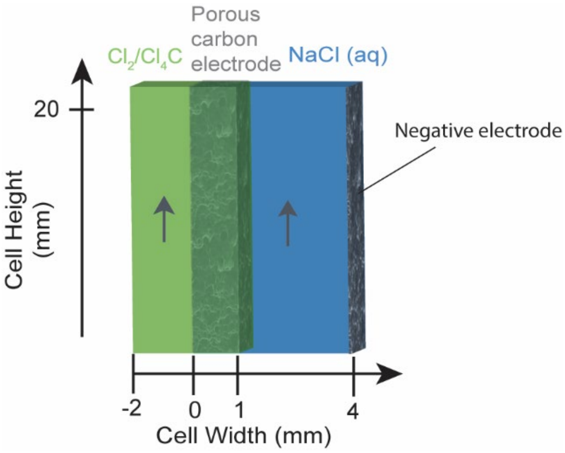

The model was built and solved in COMSOL Multiphysics 5.0 platform. In the model, the battery was divided into three sections: NaCl/H_2O (cell width= 1.0 to 4.0 mm), porous electrode (cell width= 0-1.0 mm) and the CCl_4 (arc length= -2.0 to 0 mm). In the void volume of the porous electrode, both NaCl/H_2O (aqueous phase) and CCl_4 (organic phase) are present, and Cl_2 can transfer between the two phases. The disproportionation reaction of Cl_2 in the aqueous phase was suppressed due to the high concentration of Cl^-. A continuous CCl_4 phase is needed to carry Cl_2 for redox reaction without interfering with the ion transport in the aqueous phase; the flow rates chosen in this study are below the transition to a slug flow[1] in the cell. If the two phases disperse inside the porous electrode, the particle size was assumed to be the same order of the median pore size in the carbon (10 nm according to the BET measurement), the flow pattern was evaluated by two parameters: $X = \frac{R}{L}|1 - \frac{m_p}{\rho cv}|$ and $Y = \left|1 - \frac{m_p}{\rho cv}\right|/(1 + \frac{2m_p}{\rho cv})$, in

which R is the radius of liquid particle, L is the cell height, m_p is particle mass, ρ is density, c is concentration, v is volume of the particle. X<<Y*Re (Re=Reynold's number), thus the relative motion between the two fluids is negligible[1] . The biphasic flow was treated as a phase consisting of NaCl/H_2O and CCl_4 with a specific volume ratio. To determine the physical properties of the pseudo-single phase in the porous electrode, saturation (S_j) was introduced for phase j (j=NaCl/H_2O or CCl_4). The S_j is the volumetric ratio of phase j in the void volume of the porous electrode, which was measured by displacement experiment upon flow (see next section). The Q_j is the volumetric flow rate of phase j in the cell, and the flow rate v_j is defined as follows:

$$v_j = \frac{S_j \times Q_j}{\sum S_j \times Q_j}$$

The flow rate of the pseudo-single phase is:

$$v = v_{org} * S_{org} + v_{aq} * S_{aq}$$

The overall concentration C of species i (i= Cl_2) in the pseudo-single phase in the porous electrode is:

$$C_i = \sum S_j \times C_{i,j}$$

The effective diffusivity of Cl_2 in the pseudo-single phase is corrected as the harmonic average of that in the aqueous and organic phase, considering disordered mixture[2]:

$$D_{Cl2,eff} = \frac{D_{Cl2,org} \times D_{Cl2,aq}}{\left(1 - S_{org}\right) \times D_{Cl2,org} + S_{org} \times D_{Cl2,aq}}$$

Since Cl^- and Na^+ are only present in the aqueous phase, the diffusivity and conductivity will only be discussed as in the aqueous phase. The diffusivity and conductivity of Cl^-, $Na^{+,}$ and Cl_2 were modified using Buggerman correlation[3] to account for the tortuosity of the porous electrode.

Due to the significantly larger size of the reservoir than the flow cell, the steady-state model was developed at the designated state of charge. Nernst-Plank equation was solved in both the porous electrode and NaCl/H_2O to account for the species distribution. The porous electrode was treated as the superimposition of the mixed liquid phase and the carbon network as in porous electrode theory[4, 5]. Since the reaction only occurs at the NaCl/H_2O-carbon interface, the reaction area is corrected by the fractional wettability X_j of NaCl/H_2O in the electrode. In the organic phase, only Cl_2 is soluble, and the transport of Cl_2 obeys Fick's law.

The negative electrode is grounded, the cell potential equals the sum of the equilibrium potential of the positive electrode and the overpotential of both electrodes.

$$E_{cell} = E_{eq,pos} + \eta_{pos} + \eta_{neg}$$

The equilibrium potential of the positive electrode is determined by the Nernst equation and the local concentration of Cl^- and Cl_2 in the aqueous phase.

$$E_{eq,pos} = E_{pos}^{0} + \frac{RT}{2 \times F} ln \frac{c_{Cl_2,aq}}{\left(c_{Cl^-,aq}\right)^2}$$

The overpotential is related to the local current density by Bulter-Volmer kinetics:

$$i_{loc} = i_0 \left[exp\left(\frac{\alpha F\eta}{RT}\right) - exp\left(-\frac{(1-\alpha)F\eta}{RT}\right)\right]$$

The total current density on the electrode:

$$i_{tot} = \int_0^h i_{loc} dh$$

There are two charge carriers: electrons and ions in the porous electrode, the electrical and ionic currents are denoted as i_e and i_{ion}, respectively. The corresponding potentials are $\emptyset_{\boldsymbol{e}}$ and $\emptyset_{\boldsymbol{ion}}$.

The volume percentage of CCl_4 and NaCl/H_2O in the porous electrode

The wettabilities of the porous electrode to NaCl/H_2O and CCl_4 was characterized by displacement experiment:

A dry porous carbon electrode was weighted (M_0), and then we allowed a NaCl/H_2O stream flow through the electrode and obtained the weight of the NaCl/H_2O soaked electrode (M_1). Afterward, we allowed a stream of carbon tetrachloride to flow through the NaCl/H_2O soaked electrode for liquid displacement, then weighed the electrode and got the value M_2. This weight should equal the summation of M_0 add M_{a1} (remained aqueous solution after CCl_4 flow through the electrode) add M_{CCl4} (the weight of CCl_4 into the pores of electrode). The M_{a2} is the weight of the aqueous solution replaced by the CCl_4. Here we assume the volume of the aqueous solution replaced by the carbon tetrachloride is equal.

So we can get: This weight should equal to the original porous electrode weight (M_0) add the weight of aqueous electrolyte in the pores of electrode (M_{a1} + M_{a2})$M_1 = M_0 + M_{a1} + M_{a2}$; $M_2 = M_0 + M_{a1} + M_{CCl4}$; $M_{CCl4}/\rho_{CCl4} = M_{a2}/\rho_{aq}$

According to the density table and the weight data:

ρ_{CCl4} = 1.584 g/cm^3; ρ_{aq} = 1.33 g/cm^3; M_0 =117 mg; M_1 =140 mg; M_2 = 148mg

We can calculate that the M_{a1}=9.3mg and 0.007cm^3 M_{CCl4}= 21.7 mg and 0.0137 cm^3. So, the volume ratio of the aqueous solution to the carbon tetrachloride during the flowing process is 0.007/0.0137=0.51. The volume percent of the aqueous solution in the porous electrode is 33.8%, the carbon tetrachloride is 66.2%.

Table S1. The governing equations and boundary conditions of the model cell

Section	Governing equations	Boundary conditions
NaCl/H_2O<br>(i= Na^+, Cl^-, j= aqueous phase)	$\boldsymbol{\nabla \cdot i_{ion} = 0}$<br>$\boldsymbol{i_{ion} = F \sum z_i N_i}$<br>$\boldsymbol{0 = \nabla \cdot N_i - R_i}$	**<u>At x = 4 mm</u>**<br>$0 = -n \cdot N_i$<br>$R_i = \boldsymbol{i_{loc} m_i}$

	$$\boldsymbol{N_i = -D_i \nabla c_i - z_i u_i F c_i \nabla \emptyset_{ion} + v c_i}$$ $$\boldsymbol{0 = \sum z_i c_i}$$ N_i = molar flux of species i; R_i = change of molar flux of i due to reactions	$$\int i_{loc}\, dh = -i_{avg} \int dh$$ **<u>At y = 0 mm</u>** $$v \cdot N_i = n \cdot (v \times c0_{i,j})$$ **<u>At y = 20 mm</u>** $$-n \cdot D_i \nabla c_i = 0$$
Porous electrode (i=Na^+, Cl^- and Cl_2, j=organic and aqueous phase, the diffusivity of all species are effective diffusivity in the porous electrode)	$$\boldsymbol{\nabla \cdot i_{ion} = F \sum z_i R_i}$$ $$\boldsymbol{\nabla \cdot i_{ion} + \nabla \cdot i_e = i_{tot}}$$ $$\boldsymbol{i_e = -\sigma_e \nabla \emptyset_e}$$ $$\boldsymbol{i_{ion} = F \sum z_i N_i}$$ $$\boldsymbol{0 = \nabla \cdot N_i - R_i}$$ $$\boldsymbol{N_i = -D_i \nabla c_i - z_i u_i F c_i \nabla \emptyset_{ion} + v c_i}$$ $$\boldsymbol{R_i = i_{loc} m_i}$$ $$\boldsymbol{\eta_{pos} = \phi_e - \phi_{ion} - E_{eq,pos}}$$ $$\boldsymbol{0 = \sum z_i c_i}$$ N_i = molar flux of i; R_i = change of molar flux of i due to reactions	**<u>At x = 0 mm</u>** $$\int i_{loc} dh = i_{avg} \int dh$$ **<u>At y = 0 mm</u>** $$v \cdot N_i = n \cdot (v \times c0_i)$$ **<u>At y = 20 mm</u>** $$-n \cdot D_i \nabla c_i = 0$$
Organic phase (i=Cl_2, j=organic phase)	$$\boldsymbol{0 = \nabla \cdot N_i}$$ $$\boldsymbol{N_i = -D_i \nabla c_i + v c_i}$$	**<u>At x = 0 mm</u>** $$c_{Cl2,org} = \frac{K \times c_{Cl2}}{K \times S_{org} + S_{aq}}$$ **<u>At x = -2 mm</u>** $$0 = -n \cdot N_i$$ **<u>At y = 20 mm</u>** $$v \cdot N_i = n \cdot (v \times c0_{i,j})$$

Table S2. The initial condition of the cell

Section	Initial conditions (Concentrations At y% SOC)
**CCl_4**	$c_{cl2,org}$=2 mol/L * y%
**$NaCl/H_2O$**	$c_{Na+,aq} = c_{Cl-,aq} = 6.60 * (1 - y\%)$ mol/L<br>$c_{cl2,aq} = c_{cl2,org} * 0.00272$
**Porous electrode**	$c_{cl2}=66.2\%*c_{cl2,org}+33.8\%*c_{cl2,aq}$

Table S3. The parameters and constants used in the model

Parameters/ Constants	Value/Expression	Physical meaning	Reference/ measurement
T	298.15 [K]	Temperature	
h_{cell}	20 [mm]	Cell height	
w_{cell}	4 [mm]	Cell width	
w_{pos}	1 [mm]	Positive electrode width	
l_{cell}	10 [mm]	Cell length	
$D_{Na+,aq}$	$7.96*10^{-10}$ [m²/s]	Diffusivity of Na^+ in NaCl solution	[6]
$D_{Cl-,aq}$	$1.06*10^{-9}$ [m²/s]	Diffusivity of Cl^- in NaCl solution	[6]
$D_{Cl2,aq}$	$1.38*10^{-9}$ [m²/s]	Diffusivity of Cl_2 in NaCl solution	[7]
$D_{Cl2,org}$	$3.06*10^{-9}$ [m²/s]	Diffusivity of Cl_2 in CCl_4	[8]
$D_{Cl2,eff}$	$D_{Cl2,org}*D_{Cl2,aq}/((1-S_{org})*D_{Cl2,org}+S_{org}*D_{Cl2,aq})$	Effective diffusivity of the Cl_2 in the pseudo-single phase in the porous electrode	
σ_e	0.189 [S/m]	Electrode conductivity	Resistant measurement
K	368	Partition coefficient of Cl_2 between CCl_4 and NaCl/H_2O	Estimated by the solubility[9]
a_{carbon}	911.4205 [m²/g]	Surface area of the porous carbon	BET test
a_{pos}	$a_{carbon}S_{aq}/\rho_{pos}$ = $2.9401*10^{-8}$ [m^{-1}]	Reaction area in the porous electrode	
ε	0.6	Porosity of the carbon	
ρ_{pos}	3.1 [cm³/g]	Tapped density of the positive electrode	
k_{pos}	$1.24*10^{-7}$ [cm/s]	Rate constant of positive electrode	[10]
i_{neg}^0	20 [mA/cm²]	Exchange current density of the negative electrode	Tafel plot
α_{neg}	0.57	Transfer coefficient of the negative electrode	Tafel plot

α_{pos}	0.5	Transfer coefficient of the positive electrode	[10]
S_{org}	0.663	Saturation of organic phase	Displacement experiment
S_{aq}	0.337	Saturation of aqueous phase	Displacement experiment
E_{pos}^{0}	1.358 V vs. NHE	Standard equilibrium potential of Cl_2/Cl^-	Cyclic Voltammetry
$E_{eq,neg}$	-0.5V vs NHE	Equilibrium potential of the negative electrode	Cyclic Voltammetry
m_{Cl2}	-0.5	Stoichiometric coefficient of the species for each election transfer	
m_{Na+}	1		
m_{Cl-}	1		
z_{Cl-}	-1	Charge of the species	
z_{Na+}	+1		
F	96485 [sA/mol]	Faraday constant	
n		the outward unit normal vector	

Table S4. Dependent variables

Variables	Physical meanings
i_{tot}	Current density [mA/cm^2]
Q_{aq}	Volumetric flow rate of the aqueous phase [mL/s]
Q_{org}	Volumetric flow rate of the organic phase [mL/s]

Supplementary Figures

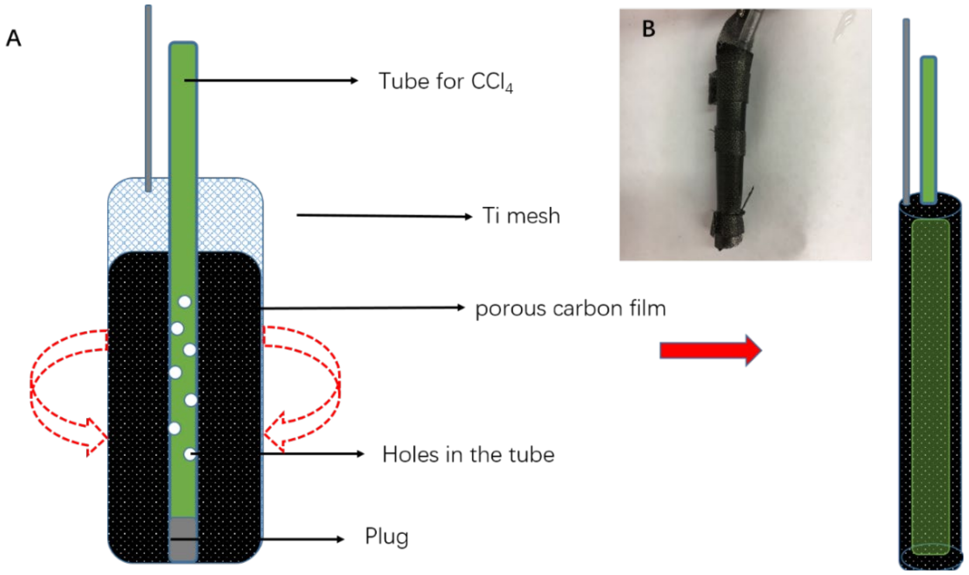

Fig. S1. Schematic and picture of the positive electrode. (A) Schematic of the positive electrode. (B) Picture of the positive electrode. The titanium grid was applied as the current collector. The porous carbon film was prepared and pressed onto the titanium grid at 10 MPa. Then we wind this electrode with a tube inside it. The tube was plugged at the end and bore some holes in the tube, enabling the carbon tetrachloride flow to spurt on the surface of the porous carbon electrode. The size of the porous carbon electrode (RuO_2-TiO_2@C) is 1.0 mm-thick and 2.0 cm^2 area. The inner diameter of the tube containing CCl_4 and RuO2-TiO2@C working electrode is 2.0 mm, the thickness of the RuO_2-TiO_2@C electrode is 1.0 mm, the distance between the working and counter electrode is 3.0 mm, and the thickness of the counter electrode is 3 mm. The height of the cell is 2.0 cm, and the volume capacity of the cell is around 2 mL. The total volumes of the CCl_4 reservoir and the NaCl/H_2O reservoir are 6.0 mL and 2.0 mL, respectively.

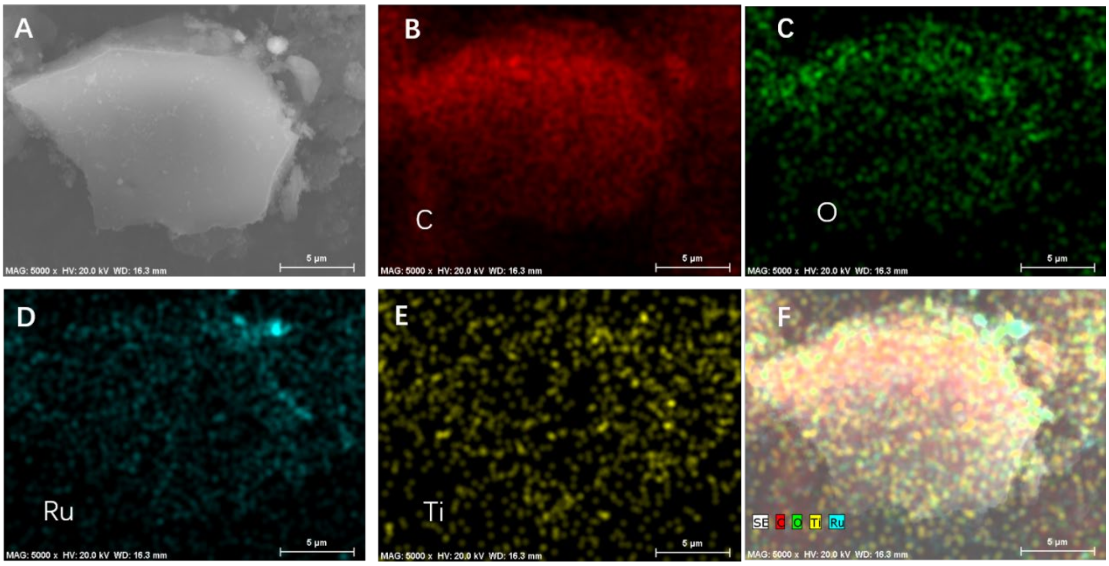

Fig. S2. Elemental mapping of as activated carbon with catalytic particles. (A) Scanning electron microscopy (SEM) images, and **(B-F)** energy dispersive X-ray spectra of **(B)** carbon, **(C)** oxygen, **(D)** ruthenium, **(E)** titanium, and **(F)** overlay of all elements. The elemental mapping of the as-prepared activated carbon with catalytic particles anchored on the surface indicates that the elements of Ru, Ti, and O are homogeneously distributed.

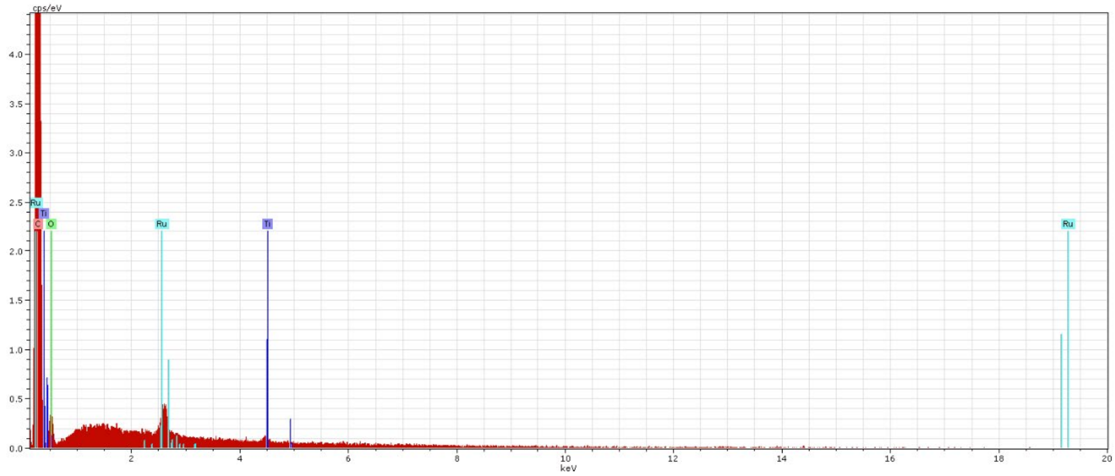

Fig. S3. Energy Dispersive X-ray spectra (EDX) mapping of the activated carbon with catalytic particles. EDX analysis shows the presence of Ru and Ti anchored on the activated carbon.

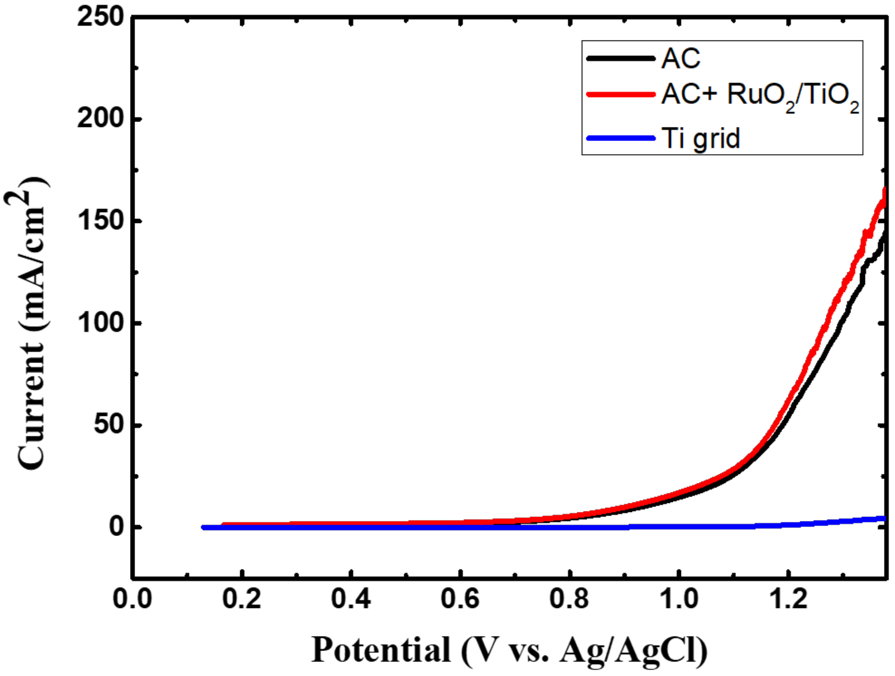

Fig. S4. Linear sweep voltammetry (LSV) from different anodes in saturated NaCl aqueous solution at a sweep rate of 5 mV/s. In Fig. S4, the onset potentials of chlorine evolution reaction (CER) shift slightly toward the negative direction in the order of Ti grid > AC (bare activated carbon) > AC+ RuO_2/TiO_2 (activated carbon anchored with RuO_2/TiO_2 particles), which shows a good CER capability of the as-prepared porous carbon electrode.

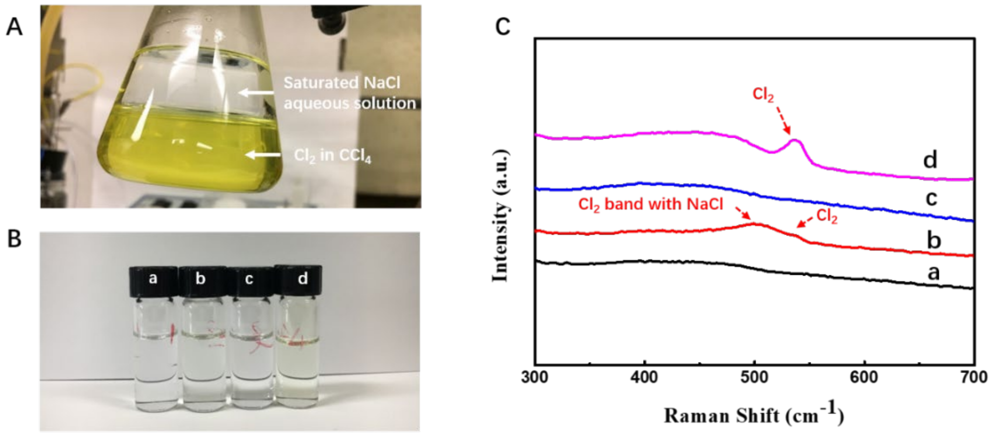

Fig. S5. Pictures of different solutions with chlorine and Raman spectrums. (A) Picture of the saturated NaCl aqueous solution and Cl_2 dissolved in the CCl_4 phase. The two phases are immiscible. **(B)** Pictures of four different solutions: (a) NaCl aqueous solution, (b) NaCl aqueous solution after bubbled with Cl_2 gas for 5 minutes, (c) NaCl aqueous solution after 60 minutes discharging process as shown in Fig.1A, and (d) water after bubbled with Cl_2 gas for 5 minutes. **(C)** Raman spectrums of the four liquid samples in Fig. S5B.

The peak at 540 cm^{-1} in Fig. S5C-d is assigned to be chlorine[11], and the peak at 500 cm^{-1} and 540 cm^{-1} in Fig. S5C-b are also assigned to chlorine[12], which indicates that trace chlorine dissolves in the NaCl aqueous solution although the solubility is very low. However, the Raman spectrum (Fig.S5C-c) of NaCl aqueous solution after 60 minutes of discharging process does not show an obvious Cl_2 peak, which indicates CCl_4 stops the dissolution of Cl_2 into NaCl/H_2O.

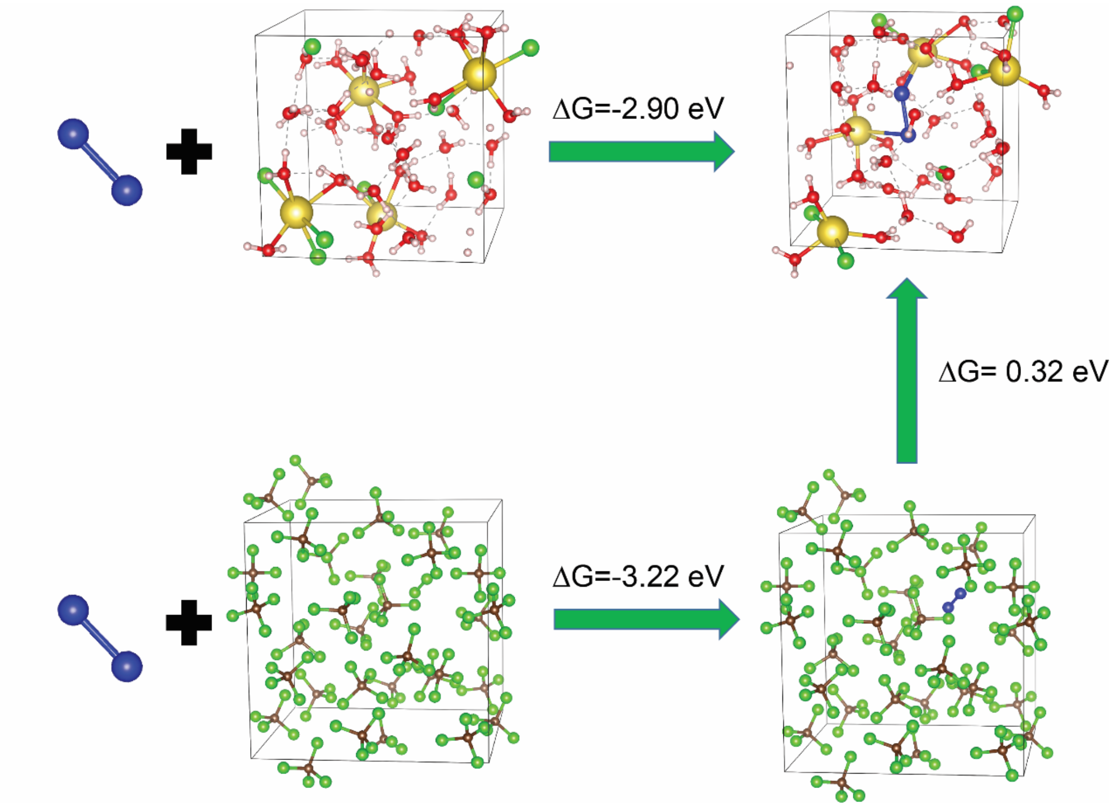

Fig. S6. Difference of solvation energy of Cl_2 in (A) CCl_4 and (B) saturated NaCl solution calculated by Ab initio Molecule Dynamic. The blue balls represent the Cl atom in Cl2, and the green, brown, red, white, and yellow balls represent the Cl, C, O, H, and Na atoms, respectively.

The result indicates that Cl_2 in CCl_4 does not spontaneously transfer to NaCl solution due to a positive Gibbs free energy. The reason is due to the unfavorable displacement of Cl^- from Na^+ when Cl_2 enters NaCl solution. The AIMD calculation was performed with the VASP package[13-15]. The ion-electron interaction is described with the Projector Augmented Wave (PAW) method, and the exchange-correlation energy is described by the functional of the Perdew–Burke–Ernzerhof (PBE) form of the generalized gradient approximation (GGA)[16-18]. Plane wave energy cut-off of 350 eV is chosen, and a minimal Γ-centered 1 x 1 x 1 k-point grid is used. All molecular dynamics simulations were performed in the NVT ensemble using a Nosé−Hoover thermostat. Each system was heated to 300 K, equilibrated for 10.0 ps, and then simulated for 10.0 ps to get the average free energy. The visualization of the structures is made by using VESTA software[19].

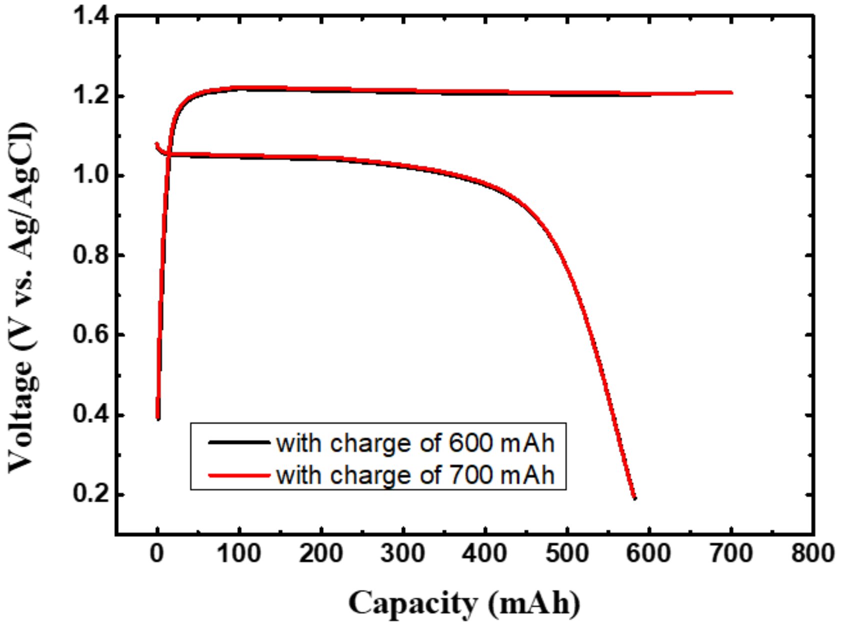

Fig. S7. Galvanostatic charge and discharge profiles of liquid flow Cl_2-CCl_4 cathode at the rate of 20 mA/cm^2 with a constant charge storage capacity of 600 mAh and 700 mAh, Q_{aq}(flow rate of NaCl solution)=0.02 mL/s and Q_{org}(flow rate of CCl_4)=0.002 mL/s.

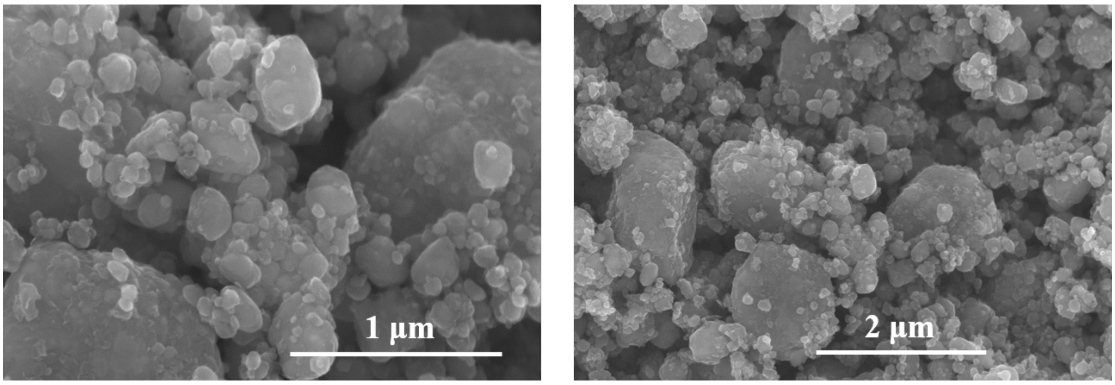

Fig. S8. Scanning electron microscopy (SEM) images of as-prepared $NaTi_2(PO_4)_3$.

Scanning electron microscopy (SEM) images of the carbon-coated $NaTi_2(PO_4)_3$ are shown in Fig. S8. The agglomerate size is close to 1–2 µm, but the crystal size is much smaller than the agglomerate size. The average individual particles size of carbon-coated $NaTi_2(PO_4)_3$ is100-200 nm.

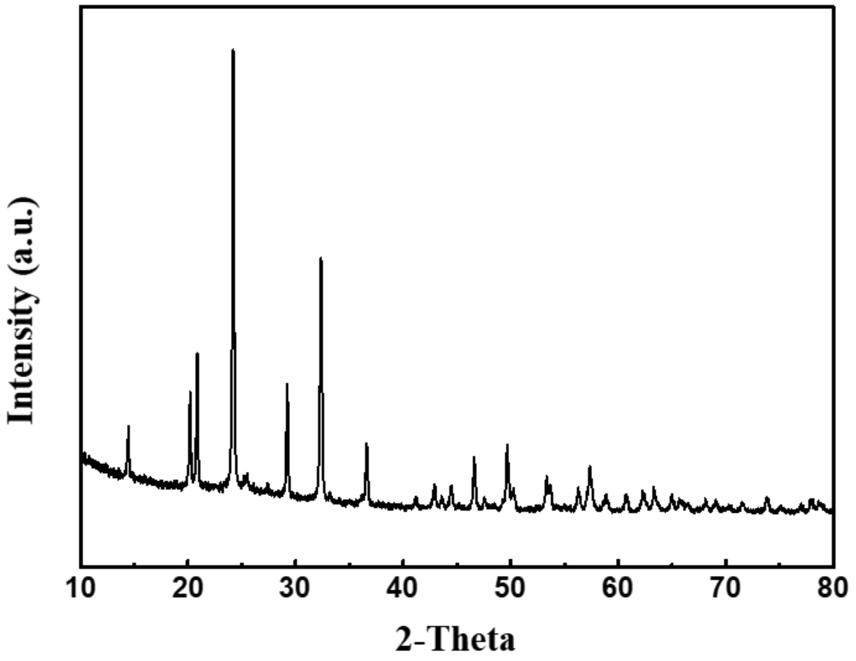

Fig. S9. X-ray diffraction (XRD) patterns of the carbon-coated $NaTi_2(PO_4)_3$. The X-ray diffraction (XRD) patterns of the carbon-coated $NaTi_2(PO_4)_3$ agree with previously reported data for this material[20].

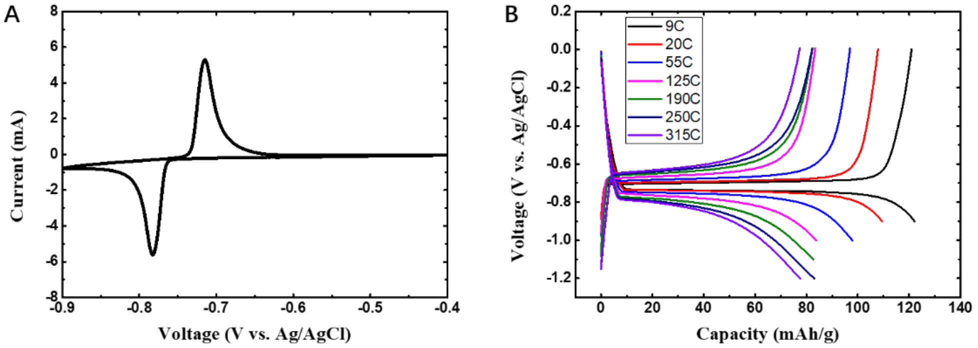

Fig. S10. Electrochemical performance of carbon-coated $NaTi_2(PO_4)_3$. (**A**) Cyclic Voltammograms for carbon-coated $NaTi_2(PO_4)_3$ in NaCl/H_2O at 0.2 mV s^{-1}; (**B**) Rate performance of carbon-coated $NaTi_2(PO_4)_3$ in saturated NaCl aqueous solution.

Fig. S10A shows the Cyclic Voltammograms for carbon-coated $NaTi_2(PO_4)_3$ in saturated NaCl aqueous solution. The anodic and cathodic peaks mirror each other, indicating the good reversibility of the material. Fig. S10B shows the rate performance of carbon-coated $NaTi_2(PO_4)_3$. Even at the rate of 315 C, the specific capacity can remain 80 mAh g^{-1} (65% at 9C).

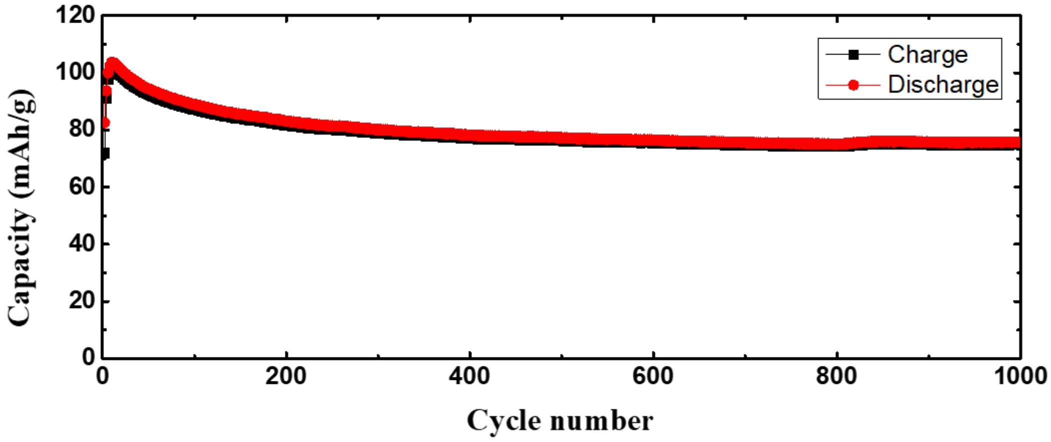

Fig. S11. Cycle performance of carbon-coated $NaTi_2(PO_4)_3$ in saturated NaCl electrolyte at the rate of 20 C.

Fig. S11 shows the cycle performance of carbon-coated $NaTi_2(PO_4)_3$ in saturated NaCl solution at 20 C rate. This material can deliver good capacity retention of 80% after 1000 cycles.

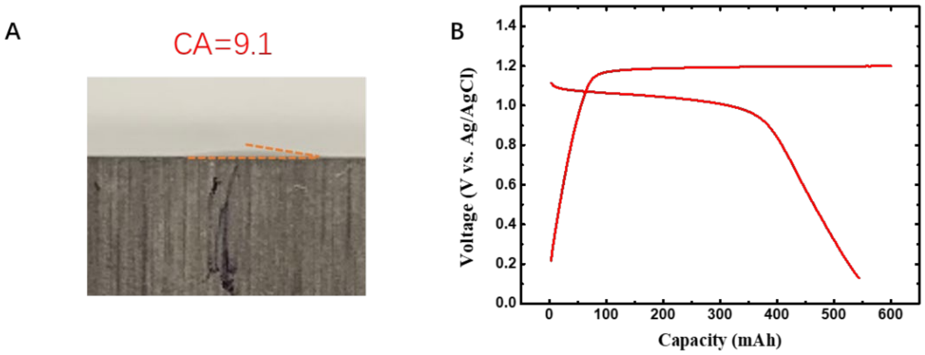

Fig. S12. Galvanostatic charge and discharge profiles of liquid Cl_2-mineral spirits cathode. (**A**) Contact angle (CA) of mineral spirits on graphite plate electrode. (**B**) Galvanostatic charge/discharge profiles of flow liquid Cl_2-mineral spirits at 20 mA/cm^2 with a constant charge storage capacity of 600 mAh and reversible discharge capacity of 550 mAh. The volume of mineral spirit is 6 mL, rendering a volumetric capacity of 91.6 Ah/L. The size of the porous RuO_2-TiO_2@C electrode is 1.0 mm -thick and 2.0 cm^2 area.

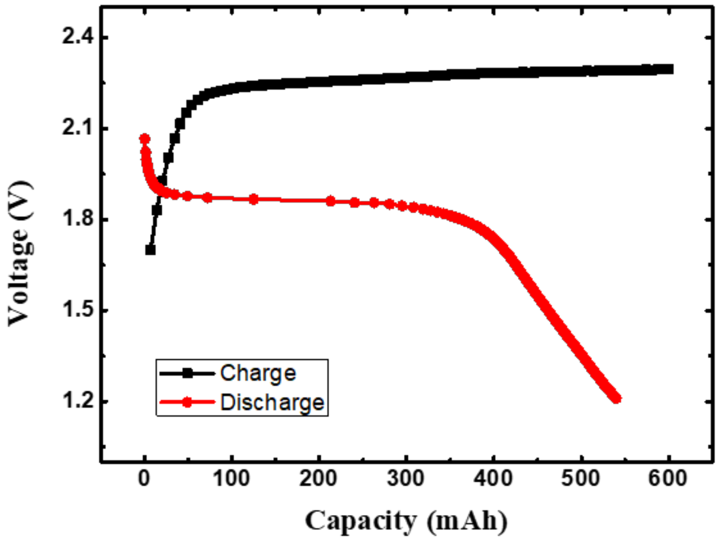

Fig. S13. Electrochemical performance of Zn//Cl_2-CCl_4 flow battery Galvanostatic Charge/discharge profiles of Zn//Cl_2-CCl_4 flow battery with the aqueous electrolyte (4M $ZnCl_2$+ 2M NaCl) at 50 mA/cm^2 with a constant charge storage capacity of 600 mAh. The amount of CCl_4 is 6 mL, the dimensions of the porous carbon (RuO_2-TiO_2@C) is 1.0 mm-thick and 2.0 cm^2 area.

The Zn//Cl_2-CCl_4 flow battery also delivers a good electrochemical performance. Its operation voltage is around 1.9V at 50 mA/cm^2, higher than the $NaTi_2(PO_4)_3$||Cl_2 battery system (around 1.676V at the same current density).

Table S5. The performance and cost comparison of Redox Flow battery reported in the past ten years.

Anode/Cathode	*Supporting Electrolyte*	*Material costs ($/kWh)*	*Membrane & Price*	*Operating Current density (mA/cm^2)*	*Discharge voltage (V)*	*Energy density (Wh/L)*	*Energy efficiency*	*Peak power density (W/cm^2)*	*Reference*
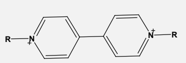 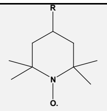	NaCl solution	~90	Dialysis membrane $20-100/m^2	40-100	1.15	10	80% (20 mA/cm^2)	N/A	[21]
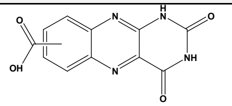 $[Fe(CN)_6]^{3-}$	KOH solution	N/A	Nafion $500/m^2	0-600	1.15	129.6	<70% (100 mA/cm^2)	0.35	[22]
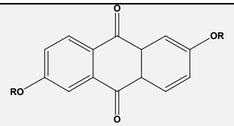 $[Fe(CN)_6]^{3-}$	KOH solution	~50	Fumasep E-600 (Sulfonated polyaryletherketone-copolymer)	0-300	1.05	12	~85% (100 mA/cm^2)	0.24	[23]
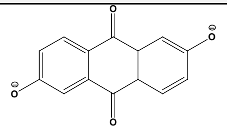 $[Fe(CN)_6]^{3-}$	KOH solution	~50	Nafion $500/m^2	0-800	1.2	6.8	84% (100 mA/cm^2)	0.4	[24]
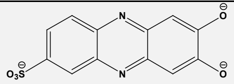 $[Fe(CN)_6]^{3-}$	NaOH solution	N/A	Nafion $500/m^2	0-100	1.4	93.8	75%-80% (100 mA/cm^2)	N/A	[25]
Zn I_3^-	3.5 M or 5.0 M ZnI_2 solution	50-98	Nafion 115 $500/m^2	5-30	1.2	~166.7	82% (10mA/cm^2)	N/A	[26]
H_2, 5M Br_2	3.5 M HBr	18.2-36.2	Membrane free	0-1600	1.05	N/A	~1% (no separation at the outlet) 66% (separation	0.795	[27]

							at the outlet)		
V^{3+} VO^{2+}	HCl	90	Nafion 112<br>$500/m^2	0-100	1.4	22.6-43.1	88-90%<br>(25 mA/cm^2)	N/A	28
V^{3+} VO^{2+}	N/A	N/A	N/A	0-1800	1.4	N/A	N/A	0.5	29
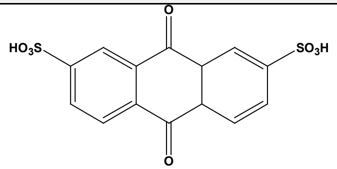	HBr	N/A	Nafion<br>$500/m^2	200-500	0.7	16	~72%<br>(200 mA/cm^2)	0.6	30
Li I_2	1M $LiPF_6$ EC/DMC (3/7 v/v) @ negative electrode (unknown amount); 0.5 M LiI (theoretically 8.2 M) @ positive electrode	34.3-45.1	LATP (Li_2O-Al_2O_3 –TiO_2 –P_2O_5)<br>$612,500/m^2	1.25-50	3.5	30<br>(Theoretically 500)	N/A	0.13	31
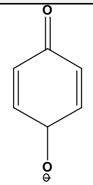 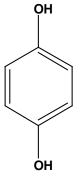	HCl	N/A	Membrane free	0-0.4	1.4	22.5	70%<br>(0.2 mA/cm^2)	0.0006	32
Zn *Br_2*	2M $ZnBr_2$, 0.1 M<br>N-methyl-ethyl-morpholinium	15-20	Nafion 115<br>$500/m^2	20-40	1.6	60-70	82%<br>(20 mA/cm^2)	0.07	33
Li *$LiFePO_4$*	1M $LiPF_6$ EC/DMC (1:1 w/w)	~40	Celgard 2500<br>$10-20/m^2	0.1-0.6	3.4	67	90%	0.328	34
This work<br>***$NaTi_2(PO_4)_3$*** ***Cl_2/Cl^-***	6 M NaCl solution	~5	Membrane free	0-200	1.76	125.7 (20℃)	91%<br>(10 mA/cm^2)	0.325	

According to the cost evaluations for all-vanadium flow batteries, the active materials take up 37% of the total cost, and the membranes take up ~30% of the total cost, electrolyte storage takes up 8%, the pump and heat exchange takes up 3%, and other operational cost takes up 21%[35]. Thus, only active material and membrane costs were compared here as other costs are unavailable for most systems. The cost/kWh includes only the electrolyte and active materials used for cell assembly. The prices for mineral commodities: NaCl (~$40/ton), Zn ($2090.34/ton), I_2 ($18500-$32000/ton), Br_2 ($4400/ton), Li metal ($121600/ton), V_2O_5 ($5568-$9300/ton) were collected from National Minerals Information Center[36]. The prices for other commodities: ZnI_2 ($5-10/kg), $ZnBr_2$ ($3-5/kg), CCl_4 ($1000/ton) $LiFePO_4$ ($8100/ton) were collected from Alibaba. The price of $NH_4H_2PO_4$ ($300/ton), TiO_2 ($1000/ton), Na_2CO_3 ($200/ton) for $NaTi_2(PO_4)_3$ anode were also collected from Alibaba[37]. The prices for organic redox couples were projectiles for mass production provided by the corresponding references. The unit cost of Nafion membranes[3838] and LATP electrolyte[39] were listed separately.

Supplementary Notes 2

Evaluation of chlorine permeation from the battery system

The cell is a closed system with the Cl_2-CCl_4 in a glass container assembled under atmospheric pressure. Therefore, the leakage could potentially occur at the seal through either chemical corrosion or permeation driven by the pressure difference across the sealing gasket. The sealing gasket for the bottle and the tubing for Cl_2-CCl_4 flow consists of a Viton fluoroelastomer. The Viton fluoroelastomer is resistant to chlorine corrosion. It is also resistant to swelling by halogenated solvents, demonstrating less than 10% volume expansion[40] in a wide temperature range between -20 ºC to 200 ºC. Thus, we only consider the leakage through Cl_2 permeation.

Cl_2 permeation rate was calculated with equation S2[41], which depends on the difference of Cl_2 partial pressure (p_{Cl2}) inside and outside the gasket. The partial pressure of Cl_2 for Cl_2-CCl_4 at different concentrations can be calculated with Raoult's law for the binary mixture. The p_{Cl2} inside the container is 111.22 kPa when concentration of Cl_2 is 0.184 mol/mol CCl_4 (saturation) at 20 ºC . At 50 ºC, the p_{Cl2} of saturated Cl_2-CCl_4 increases to 116.0 kPa (0.088 mol Cl_2/mol CCl_4) (Table S*6*). The p_{Cl2} outside the container is assumed to be 0 kPa. The permeation flux of Cl_2 through the Viton gasket (F) into the atmosphere calculated through equation S2 is between 1.24 to 19.03 mL/day (3.72- 57.1 mg/day) at 20 ºC and 2.08 to 30.60 mL/day (5.1- 75 mg/day) at 50 ºC (The variations are caused by the different permeation coefficients reported for different fluoroelastomers[42]*)*. At these leakage rates, it requires 0.5 to 20 days at 20 ºC or 0.3 to 15 days at 50 ºC to reach the permissible exposure limit (PEL) of chlorine (3mg/ m^3 [43]) in an unventilated 1 m^2 * 2 m storage space (around the size of a fume hood). Thus, it is very unlikely to be exposed to chlorine higher than the PEL with the appropriate gaskets, chlorine sensor, and in a ventilated environment.

$$F = K\frac{A(p_{Cl2,in} - p_{Cl2,out})}{d} \tag{S2}$$

F= permeation flux of the gas (mL/day) ; K= permeation coefficient=60- 1000 (20 ºC), 100 -2000 (50 ºC) (mL/100 in^2/day/atm[42]) ; A= gasket surface area= (1.5 cm*1.5 cm-0.5 cm* 0.5 cm)*π*2 =12.56 cm^2; d= gasket thickness= 1 cm; $p_{Cl2,in}$ = partial pressure of Cl_2 inside the container; $p_{Cl2,out}$= partial pressure of Cl_2 outside the container

Table S6. Vapor pressure of Cl_2, CCl_4, and Cl_2-CCl_4 at 20 ºC and 50 ºC (atm).

Pressure (kPa)	P_{Cl2} (kPa)	P_{CCl4} (kPa)	p_{Cl2} (kPa) (Based on Raoult's law)
20 ºC (1 atm)	604.50[44]	13.33[45]	$p_{Cl2} = P_{Cl2} \times x_{Cl2} = 111.22$ ($x_{Cl2} \leqslant 0.184$)
50 ºC (1 atm)	1318.97[44]	53.33[45]	$p_{Cl2} = P_{Cl2} \times x_{Cl2} = 116.0$ ($x_{Cl2} \leqslant 0.088$)

Supplementary References

1. Brennen, C. E. *Fundamentals of Multiphase Flow*. 127-154 (Cambridge University Press, 2005).

2. Yang, W. W., Zhao, T. S. A Two-Dimensional, Two-Phase Mass Transport Model for Liquid-Feed DMFCs. *Electrochim. Acta* **52**, 6125–6140 (2007).

3. Tjaden, B., Cooper, S. J., Brett, D. J., Kramer, D., Shearing, P. R. On the Origin and Application of the Bruggeman Correlation for Analysing Transport Phenomena in Electrochemical Systems. *Curr. Opin. Chem. Eng.* **12**, 44–51 (2016).

4. Newman, J., Tiedemann, W. Porous-electrode Theory with Battery Applications. Porous-electrode Theory with Battery Applications. *AIChE J.* **21**, 25–41 (1975).

5. Lai, W., Ciucci, F. Mathematical Modeling of Porous Battery Electrodes-Revisit of Newman's Model. *Electrochim. Acta* **56**, 4369–4377 (2011).

6. Vitagliano, V., Lyons, P. A. Diffusion Coefficients for Aqueous Solutions of Sodium Chloride and Barium Chloride. *J. Am. Chem. Soc.* **78**, 1549–1552 (1956).

7. Tang, A., Sandall, O. C. Diffusion Coefficient of Chlorine in Water at 25–60 °C. *J. Chem. Eng. Data* ***30***, 189–191 (1985).

8. Hildebrand, J. H., Lamoreaux, R. H. Diffusivity of Gases in Liquids. *Proc. Natl. Acad. Sci.* **71**, 3321–3324 (1974).

9. Young, C. L. Sulfur dioxide. Chlorine, fluorine and chlorine oxides. *solubility data series*. **12**, 333-445 (1983).

10. Kim, J. T., Jorne, J. The Kinetics of a Chlorine Graphite Electrode in the Zinc-Chlorine Battery. *J. Electrochem. Soc.* **124**, 1473-1477 (1977).

11. Aggarwal, R. L., Farrar, L. W., Cecca, S. D., Jeys, T. H. Raman spectra and cross sections of ammonia, chlorine, hydrogen sulfide, phosgene, and sulfur dioxide toxic gases in the fingerprint region 400-1400 cm^{-1}. *Aip Advances* **6**, 025310 (2016).

12. Ault, B. S., Andrews, L. Infrared and Raman spectra of the $M^{+}Cl^{3-}$ ion pairs and their chlorine–bromine counterparts isolated in argon matrices. *J. Chem. Phys.* **64**, 4853 (1976).

13. Hohenberg, P., Kohn, W. Inhomogeneous electron Gas. *Phys. Rev.* **136**, B864-B871 (1964).

14. Kresse, G., Hafner, J. Ab initio molecular dynamics for liquid metals. *Phys. Rev. B* **49**, 14251 (1994).

15. Kohn, W., Sham, L. J. Self-Consistent Equations Including Exchange and Correlation Effects. *Phys. Rev.* **140**, A1133-A1138 (1965).

16. Blöchl, P. E. Projector augmented-wave method. *Phys. Rev. B* **50**, 17953 (1994).

17. Perdew, J. P., Burke, K., Ernzerhof, M. Generalized Gradient Approximation Made Simple. *Phys. Rev. Lett.* **77**, 3865 (1996).

18. Kresse, G., Furthmüller, J. Efficient iterative schemes for ab initio total-energy calculations using a plane-wave basis set. *Phys. Rev. B* **54**, 11169 (1996).

19. Momma, K., Izumi, F. "VESTA 3 for three-dimensional visualization of crystal, volumetric and morphology data," *J. Appl. Crystallogr.* **44**, 1272-1276 (2011).

20. Chen, L., Liu, J., Guo, Z., Wang, Y., Wang, C., Xia, Y. Electrochemical Profile of $LiTi_2(PO_4)_3$ and $NaTi_2(PO_4)_3$ in lithium, sodium or mixed ion aqueous solutions. *J. Electrochem. Soc.* **163,** A904-A910 (2016).

21. Janoschka, T., Martin, N., Martin, U., Friebe, C., Morgenstern, S., Hiller, H., Hager, M. D., Schubert, U. S. An Aqueous, Polymer-Based Redox-Flow Battery Using Non-Corrosive, Safe, and Low-Cost Materials. *Nature* **527**, 78–81 (2015).

22. Lin, K., Gómez-Bombarelli, R., Beh, E. S., Tong, L., Chen, Q., Valle, A., Aspuru-Guzik, A., Aziz, M. J., Gordon, R. G. A Redox-Flow Battery with an Alloxazine-Based Organic Electrolyte. *Nat. Energy* **1**, 16102 (2016).

23. Kwabi, D. G., Lin, K., Ji, Y., Kerr, E. F., Goulet, M.-A., De Porcellinis, D., Tabor, D. P., Pollack, D. A., Aspuru-Guzik, A., Gordon, R. G. Alkaline Quinone Flow Battery with Long Lifetime at PH 12. *Joule* **2**, 1894–1906 (2018).

24. Lin, K., Chen, Q., Gerhardt, M. R., Tong, L., Kim, S. B., Eisenach, L., Valle, A. W., Hardee, D., Gordon, R. G., Aziz, M. J. Alkaline Quinone Flow Battery. *Science* **349**, 1529–1532 (2015).

25. Hollas, A., Wei, X., Murugesan, V., Nie, Z., Li, B., Reed, D., Liu, J., Sprenkle, V., Wang, W. A Biomimetic High-Capacity Phenazine-Based Anolyte for Aqueous Organic Redox Flow Batteries. *Nat. Energy* **3**, 508–514 (2018).

26. Li, B., Nie, Z., Vijayakumar, M., Li, G., Liu, J., Sprenkle, V., Wang, W. Ambipolar zinc-polyiodide electrolyte for a high-energy density aqueous redox flow battery. *Nat. Commun.* **6**, 6303 (2015).

27. Braff, W. A., Bazant, M. Z., Buie, C. R. Membrane-Less Hydrogen Bromine Flow Battery. *Nat. Commun.* **4**, 1–6 (2013).

28. Li, L., Kim, S., Wang, W., Vijayakumar, M., Nie, Z., Chen, B., Zhang, J., Xia, G., Hu, J., Graff, G., Liu, J., Yang, Z. A Stable Vanadium Redox-Flow Battery with High Energy Density for Large-Scale Energy Storage. *Adv. Energy Mater.* **1**, 394–400 (2011).

29. Perry, M. L., Darling, R. M., Zaffou, R. High Power Density Redox Flow Battery Cells. *ECS Trans.* **53**, 7−16 (2013).

30. Huskinson, B., Marshak, M. P., Suh, C., Er, S., Gerhardt, M. R., Galvin, C. J., Chen, X., Aspuru-Guzik, A., Gordon, R. G., Aziz, M. J. A Metal-Free Organic-Inorganic Aqueous Flow Battery. *Nature* **505**, 195–198 (2014).

31. Zhao, Y., Byon, H. R. High-Performance Lithium-Iodine Flow Battery. *Adv. Energy Mater.*

3, 1630–1635 (2013).

32. Navalpotro, P., Palma, J., Anderson, M., Marcilla, R. A Membrane-Free Redox Flow Battery with Two Immiscible Redox Electrolytes. *Angew. Chemie Int. Ed.* **56,** 12460–12465 (2017).

33. Lai, Q., Zhang, H., Li, X., Zhang, L., Cheng, Y. A novel single flow zinc–bromine battery with improved energy density. *J. Power Sources* **235**, 1–4 (2013).

34. Hamelet, S., Tzedakis, T., Leriche, J.-B., Sailler, S., Larcher, D., Taberna, P.-L., Simon, P. and Tarascona, J.-M. Non-Aqueous Li-Based Redox Flow Batteries. *J. Electrochem. Soc.* **159,** A1360-A1367 (2012).

35. Moore, M., Watson, J., Zawodzinski Jr., T. A., Zhang, M. & Counce, R. M. Capital Cost Sensitivity Analysis of an All-Vanadium Redox-Flow Battery. *ECS Transactions* **41**, 1–19 (2012).

36. (A) Emily K. Schnebele, "2016 Mineral Yearbook. Bromine" U.S Geological Survey. Retrieved from https://s3-us-west-2.amazonaws.com/prd-wret/assets/palladium/production/mineral-pubs/bromine/myb1-2016-bromi.pdf (B) Amy Tocin, "2016 Mineral Yearbook. Zinc" U.S Geological Survey. Retrieved from https://www.usgs.gov/centers/nmic/zinc-statistics-and-information (C) Emily K. Schnebele, "2016 Mineral Yearbook. Iodine" U.S Geological Survey. Retrieved from https://s3-us-west-2.amazonaws.com/prd-wret/assets/palladium/production/mineral-pubs/iodine/myb1-2016-iodin.pdf (D) Brain Jaskula, "2016 Mineral Yearbook. Lithium" U.S Geological Survey. Retrieved from https://s3-us-west-2.amazonaws.com/prd-wret/assets/palladium/production/mineral-pubs/lithium/myb1-2016-lithi.pdf (E) Desiree E. Polyak, "2016 Mineral Yearbook. Vanadium" U.S Geological Survey. Retrieved from https://s3-us-west-2.amazonaws.com/prd-wret/assets/palladium/production/mineral-pubs/vanadium/myb1-2016-vanad.pdf (F) Lori Apodaca, "2015 Mineral Yearbook. Sulfur" U.S Geological Survey. Retrieved from https://s3-us-west-2.amazonaws.com/prd-wret/assets/palladium/production/mineral-pubs/sulfur/myb1-2015-sulfu.pdf (G) Wallace P. Bolen, "2015 Mineral Yearbook. Salt" U.S Geological Survey. Retrieved from https://s3-us-west-2.amazonaws.com/prd-wret/assets/palladium/production/mineral-pubs/salt/myb1-2015-salt.pdf

37. (a) $NH_4H_2PO_4$ price. Retrieved from https://www.alibaba.com/showroom/nh4h2po4%252bpowder%252bprice.html; (b) TiO_2 price. Retrieved from https://www.alibaba.com/showroom/tio2+titanium+dioxide.html?fsb=y&IndexArea=product_en&CatId=&SearchText=tio2+titanium+dioxide&isGalleryList=G; (c) Na_2CO_3 price. Retrieved from https://www.alibaba.com/showroom/na2co3.html.

38. Darling, R. M., Gallagher, K. G., Kowalski, J. A., Ha, S., Brushett, F. R. Pathways to low-cost electrochemical energy storage: a comparison of aqueous and nonaqueous flow batteries. *Energy Environ. Sci.* **7**, 3459–3477 (2014).

39. LATP electrolyte price. Retrieved from https://www.msesupplies.com/products/ampcera-lisicon-polished-membrane-19mm-diameter-200um-thickness?variant=23101038755898&utm_medium=cpc&utm_source=google&utm_campaign=Google%20Shopping&_vsrefdom=adwords&gclid=EAIaIQobChMIu4PJ1O2P5AIViJyzCh32AAQoEAQYAyABEgIyI_D_BwE

40. Dupont General Chemical Resistance Guide.

https://mscrm-dupont.secure.force.com/CRG_TlargiGuide

41. Sturm, P. *et al.* Permeation of atmospheric gases through polymer O-rings used in flasks for air sampling. *J. Geophys. Res. Atmos.* **109**, D04309 (2004).

42. Argazinski, J. K., Sant'Anna, J. A. P., Tristante, M. R. Fluoropolymers for the Chemical Processing Industry Applications. (Conference paper, check citation format)

43. Occupational Safety and Health Administration (OSHA) set the permissible exposure limit data. Retrieved from https://www.osha.gov/

44. The Chlorine Manual. Sixth ed. Washington: The Chlorine Institute, INC., 2000.

45. Hildenbrand, D. L., McDonald, R. A., The Heat of Vaporization and Vapor Pressure of Carbon Tetrachloride; The Entropy from Calorimetric Data. *J. Phys. Chem.* **63**, 1521-1522 (1959).
